# A Comparative Analysis of Pediatric Emergency Department Admissions Before and During the COVID-19 Pandemic

**DOI:** 10.7759/cureus.58436

**Published:** 2024-04-17

**Authors:** Osman Fırat Çalışkan, Gül Trabzon, Ufuk Utku Güllü, Esra Gezmen Yazarlı, Ferhat Sarı, Sevcan İpek, Çiğdem El

**Affiliations:** 1 Department of Pediatrics, Faculty of Medicine, Hatay Mustafa Kemal University, Antakya, TUR

**Keywords:** children, pediatrics, emergency department, pandemic, covid-19

## Abstract

Objectives

This study aims to evaluate the outbreak’s impact on emergency services, with findings obtained from patients who applied to our pediatric emergency service before and during the pandemic period.

Methods

In this study, the Pediatric Emergency Polyclinic of Hatay Mustafa Kemal University (MKU) Department of Pediatrics was evaluated during the COVID-19 pre-pandemic period and the COVID-19 pandemic period. Demographic features, complaints, discharge situations, diagnostic groups, and diagnoses of 16,730 non-traumatic patients one month to 18 years old were compared retrospectively.

Results

Comparing the pre-pandemic period and the pandemic period, it was determined that there was a statistically significant difference in the average age of patients, age groups, admission hours, triage classification, complaints, and diagnoses seen.

Conclusion

According to the findings obtained in the study, pediatric emergency department admissions decreased significantly during the pandemic period. As a result of the pandemic measures taken, the incidence of diseases caused by infectious agents, such as respiratory tract infections, decreased. The change in pediatric emergency service habits with the pandemic highlights the importance of conducting more comprehensive epidemiological studies in terms of more efficient and effective use of pediatric emergency health services in Turkey.

## Introduction

An emergency department is "the place where the first care of patients who need urgent care in health institutions is provided" [1]. According to the Regulation on Emergency Health Services in Turkey, emergency departments in all private and public hospitals accept all admissions without discrimination [2]. An emergency patient is defined as a patient with a life-threatening condition that needs immediate intervention. Non-urgent patients are defined as patients who do not require urgent intervention or do not require intervention for several hours [3].

According to the 2008 evaluation of the Society of Pediatric Emergency and Intensive Care Medicine (SPEICM), pediatric emergency services (PES) admissions were found to comprise approximately 30% of all emergency admissions [4]. The conditions requiring urgent medical assistance in patients admitted to PES mainly include persistent high fever, severe infections, respiratory distress, severe pain, dehydration, convulsions, and severe allergic reactions. In addition, "bone fractures, dislocations, sprains, head trauma, falls, traffic accidents, burns, asthma attacks, diabetic ketoacidosis, severe complications of congenital diseases and/or metabolic diseases, poisoning, insect bites, snake and scorpion stings" are common and life-threatening causes of admission to PES [4]. Recognizing the epidemiological characteristics of patients admitted to the emergency department, planning services according to the person and time, and profiling the disease profile of emergency patients will increase the quality of health care and the satisfaction of patients and health professionals and reduce economic losses [5].

This study aims to evaluate the changing patient density and disease profile pre-COVID-19 pandemic to the onset of the pandemic in light of epidemiological data obtained from PES admissions.

## Materials and methods

The study was conducted within the framework of ethical rules and by the principles of the Declaration of Helsinki. Before the study began, ethics committee approval and institutional permission were obtained by the Hatay Mustafa Kemal University (MKU) Tayfur Ata Sökmen Faculty of Medicine Non-Interventional Clinical Research Ethics Committee with decision number 5 on August 29, 2022. Patient consent was not obtained in this study because it was retrospective.

We conducted a retrospective study evaluating non-trauma patients aged between one month and 18 years who were admitted to the Hatay MKU Department of Pediatrics Pediatric Emergency Polyclinic between March 11, 2019-March 10, 2020 (pre-COVID-19 pandemic period) and March 11, 2020-March 10, 2021 (COVID-19 pandemic period) (16,730 admissions) [6]. The World Health Organization (WHO) criteria for diagnosing COVID-19 infection during the pandemic were applied. Medical and sociodemographic information about emergency admissions was collected retrospectively by examining electronic records through the hospital information management system software. The obtained patient information included sociodemographic characteristics, mode of presentation to the emergency department, triage groups, complaints, diagnostic groups, diagnoses, admission hours, and discharge status. A cross-sectional study was conducted with the information obtained. Electronic patient records were analyzed for complaints, diagnosis groups, diagnoses, and discharge status.

Patients admitted to the pediatric emergency polyclinic were classified into the following age ranges: <1 year, 1-3 years, 4-6 years, 7-12 years, and 13-18 years. Patients included in the study were admitted one year before the COVID-19 pandemic period (March 11, 2019), which started with the first COVID-19 case in Turkey, and one year after the COVID-19 pandemic was declared (March 10, 2021).

The data collected in the study were evaluated with the Statistical Package for the Social Sciences (IBM SPSS Statistics for Windows, IBM Corp., Version 26.0, Armonk, NY) and the Microsoft Excel 2016 package program (Microsoft® Corp., Redmond, WA). The data conformity to the normal distribution was determined by skewness and kurtosis (±1) tests. In addition to descriptive statistical methods (i.e., percentage and frequency), a chi-square test was used to compare independent groups.

## Results

The number of admissions to the pediatric emergency department before the pandemic was 13,187, compared to 3,543 patients during the pandemic period. In other words, the number of patients admitted to the pediatric emergency department decreased by 73.13% with the pandemic.

**Figure 1:**
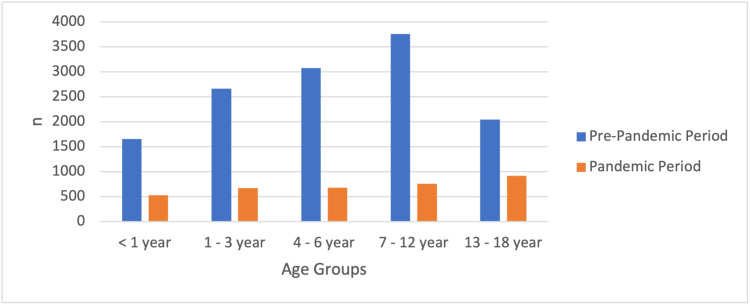
Percentage distribution of age groups according to the admission periods of the patients

The average age (in years) was 6.22 ± 4.66 in the pre-pandemic period and 7.11 ± 5.62 in the pandemic period. This indicates an average age increase during the pandemic period compared to the pre-pandemic period (p <0.05). When classified according to age group, patients admitted in the pre-pandemic period were most frequently in the "7-12 Years" age group (28.5%), while patients in the "13-18 Years" age group were most frequently admitted during the pandemic period (25.9%; Figure 1; Table 1).

**Table 1:** Average age of patients and percentage distribution of age groups according to the admission periods of the patients The data has been represented as N, %, Median. Also, included is the value at which the p-value is considered significant (p<0.05).

	Pre-pandemic Period	Pandemic Period			p
Age (years)	Median (IQR)	Median (IQR)			
	5.25 (2.16-9.66)	5.58 (2-12.25)	MWU(Z) = -5.741		0.001
Age Groups	n (%)	n (%)			
<1 year	1652 (12.5)	524 (14.8)			
1-3 years	2658 (20.2)	666 (18.8)			
4-6 years	3076 (23.3)	677 (19.1)			
7-12 years	3757 (28.5)	757 (21.4)			
13-18 years	2044 (15.5)	919 (25.9)			
Total	13187 (100)	3543 (100)	x2 = 260.07	0.001	

**Figure 2:**
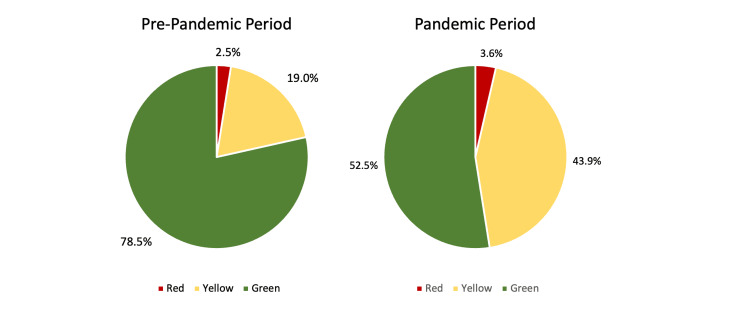
Percentage distribution of triage according to admission period Red: Patients in this category are taken to the resuscitation room and treated immediately. Yellow: Patients in this category are taken to the emergency room examination room and intervened within one hour at the latest. Green: Patients in this category are taken to the emergency department examination room and intervened within two hours at the latest.

Patients' nationality, time of admission, mode of admission, and triage status were compared between the pre-pandemic and pandemic periods. During the pandemic period, there was a significant increase in the admission of foreign national patients (p <0.05). Also, it was observed that the highest number of admissions occurred between the hours of 16:00 and 00:00, as in the pre-pandemic period. Regarding the type of admission to the pediatric emergency department, there was a significant increase in admissions by ambulance during the pandemic period compared to the pre-pandemic period (p <0.05). In the triage classification of patients at admission, a significant proportional decrease was observed in the number of patients admitted with the triage coding indicated with "Green" during the pandemic (p <0.05; Figure 2; Table 2).

**Table 2:** Distribution of patients' nationality, time of admission, mode of admission, and triage status according to admission period The data has been represented as N, %. Also, included is the value at which the p-value is considered significant (p<0.05).

	Pre-pandemic Period n = 13187 (%)	Pandemic Period n = 3543 (%)		p
Nationality			x2 = 180.897	0.001
Turkish	12121 (91.9)	2990 (84.4)		
Foreign National	1066 (8.1)	553 (15.6)		
Time of admission			x2 = 19,980	0.001
08:00-16:00	4400 (33.4)	1288 (36.4)		
16:00-00:00	7284 (55.2)	1808 (51.0)		
00:00-08:00	1503 (11.4)	447 (12.6)		
Mode of admission			x2 = 167,092	0.001
Direct admissions	12712 (96.4)	3232 (91.2)		
Ambulance	475 (3.6)	311 (8.8)		
Triage			x2 = 984,374	0.001
Red	329 (2.5)	126 (3.6)		
Yellow	2505 (19)	1556 (43.9)		
Green	10353 (78.5)	1861 (52.5)		

The most common reasons for admission to the pediatric emergency department according to admission period were compared. A significant difference was observed in 21 of the 27 reasons for admission (Table 3).

**Table 3:** Distribution of complaints of patients admitted to the pediatric emergency department according to the period of admission The data has been represented as N, %. Also, included is the value at which the p-value is considered significant (p<0.05).

	Pre-pandemic Period		Pandemic Period		p
	n	%	n	%	
Fever	4174	31.7	624	17.6	0.001
Sore throat/earache	1254	9.5	232	6.5	0.001
Headache	189	1.4	47	1.3	0.633
Dizziness	38	0.3	22	0.6	0.003
Difficulty breathing	271	2.1	133	3.8	0.001
Nosebleed	79	0.6	55	1.6	0.001
Cough	1280	9.7	177	5.0	0.001
Chest pain	100	0.8	67	1.9	0.001
Abdominal pain	973	7.4	439	12.4	0.001
Nausea/vomiting	1418	10.8	416	11.7	0.095
Constipation/diarrhea	953	7.2	176	5.0	0.001
Inguinal pain	124	0.9	86	2.4	0.001
Joint/muscle pain	145	1.1	58	1.6	0.009
Rash	459	3.5	149	4.2	0.041
Soft tissue disorders	135	1.0	66	1.9	0.001
Animal bite	112	0.8	71	2.0	0.001
Convulsion	196	1.5	107	3.0	0.001
Poisoning	167	1.3	97	2.7	0.001
Foreign body in body cavities	310	2.4	207	5.8	0.001
Toothache	130	1.0	61	1.7	0.001
General condition disorder	83	0.6	50	1.4	0.001
General medical examination	350	2.7	85	2.4	0.397
Red eyes/eye pain	149	1.1	39	1.1	0.884
Syncope	46	0.3	17	0.5	0.258
Jaundice	9	0.1	23	0.6	0.001
Vomiting blood/bloody stool	24	0.2	8	0.2	0.596
Muscle weakness/gait disorder	19	0.1	31	0.9	0.001

Patients admitted before and during the pandemic period were divided and compared according to diagnostic group. The most common diagnoses seen in these diagnostic groups were defined within each group. Aside from the most common diseases in the diagnosis group, "Infectious and Parasitic Diseases,” significant differences were observed in the diagnoses in other disease groups (p < 0.05; Table 4).

**Table 4:** Distribution of the most common diagnoses within the diagnostic groups of patients admitted to the pediatric emergency department according to period of admission The data has been represented as N, %. Also, included is the value at which the p-value is considered significant (p<0.05). URTI: upper respiratory tract infection

	Pre-pandemic Period		Pandemic Period		p
	n	%	n	%	
Infectious and Parasitic Diseases					
Infectious Diseases of the Intestines	2475	19.51	642	19.45	0.940
Viral Infections Transmitted through Skin Lesions	35	0.28	4	0.12	0.109
CNS Viral Infections	11	0.09	3	0.09	0.942
Hematologic-Oncologic System Diseases					
Hemolytic Anemias	54	0.43	39	1.18	0.001
Aplastic Anemi	21	0.17	46	1.39	0.001
Coagulation Disorders	33	0.26	18	0.55	0.010
Idiopathic Thrombocytopenic Purpura	10	0.08	11	0.33	0.001
Endocrine and Metabolic Diseases					
Metabolic Disorders	22	0.17	12	0.36	0.035
Diabetes Mellitus	8	0.06	5	0.15	0.112
Psychiatric Diseases					
Mood Disorders	6	0.05	7	0.21	0.003
Schizophrenia, Schizotypal, and Delusional Disorders	2	0.02	2	0.06	0.147
Nervous System Diseases					
Episodic and Paroxysmal Disorders	185	1.46	105	3.18	0.001
Migraine and Headache Syndromes	58	0.46	24	0.73	0.053
Intracranial Hemorrhage and Space-Occupying Lesions	44	0.35	33	1.00	0.001
CNS Inflammatory Diseases	29	0.23	6	0.18	0.608
Cerebral Palsy and Other Paralytic Diseases	11	0.09	21	0.64	0.001
Eye- and ENT-Related Diseases					
Middle Ear Diseases	196	1.55	29	0.88	0.004
Disorders of the External Auditory Canal	46	0.36	12	0.36	0.993
Conjunctival Disorders	126	0.99	35	1.06	0.730
Eyelid and Orbital Disorders	29	0.23	7	0.21	0.859
Circulatory System Diseases					
Heart Failure	52	0.41	41	1.24	0.001
Lymphadenitis	30	0.24	14	0.42	0.067
Myocarditis	5	0.04	8	0.24	0.001
Kawasaki	5	0.04	2	0.06	0.604
Respiratory System Diseases					
Acute URTI	5931	46.76	826	25.03	0.001
Acute Bronchiolitis	395	3.11	51	1.55	0.001
Pneumonia	290	2.29	82	2.48	0.500
Chronic Lower Respiratory Diseases	32	0.25	14	0.42	0.100
Digestive System Diseases					
Diseases of the Appendix	133	1.05	117	3.55	0.001
Dental Caries and Dental Abscesses	133	1.05	61	1.85	0.001
Ileus and Obstructive Bowel Diseases	75	0.59	46	1.39	0.001
Esophagus, Stomach, and Duodenal Diseases	23	0.18	36	1.09	0.001
Dermatologic System Diseases					
Urticaria	369	2.91	113	3.42	0.123
Skin and Subcutaneous Infections	45	0.35	33	1.00	0.001
Dermatitis and Eczema	32	0.25	8	0.24	0.920
Musculoskeletal System Diseases					
Soft Tissue Disorders	98	0.77	52	1.58	0.001
Inflammatory Arthropathies	23	0.18	6	0.18	0.995
Infectious Arthropathies	6	0.05	4	0.12	0.130
Osteomyelitis and Osteopathy	3	0.02	6	0.18	0.001
Nephrological and Urogenital System Diseases					
Urinary System Infections	188	1.48	75	2.27	0.001
Epididymis/Orchitis	28	0.22	21	0.64	0.001
Kidney Failure	17	0.13	11	0.33	0.015
Urolithiasis	16	0.13	9	0.27	0.058
Obstetric and Gynecological Diseases					
Pregnancy Status	1	0.01	27	0.82	0.001
Dysmenorrhea	21	0.17	16	0.48	0.001
Symptomatic Findings					
Digestive System and Abdominal Findings	491	3.87	75	2.27	0.001
Nervous and Musculoskeletal System Findings	192	1.51	92	2.79	0.001
Symptomatic Findings of the Circulatory System	65	0.51	26	0.79	0.061
Injuries and Poisoning					
Foreign Body in Body Cavities	316	2.49	210	6.36	0.001
Toxic Effects of Non-drug Substances	114	0.90	64	1.94	0.001
Bee, Insect, and Tick Stings	71	0.56	40	1.21	0.001
Poisoning with Drugs, Medicines, and Biological Substances	46	0.36	32	0.97	0.001
Scorpion Sting	37	0.29	21	0.64	0.003

Emergency department discharges (discharge, hospitalization, referral, exitus) were compared before and during the pandemic. A statistically significant difference was observed when compared according to the admission period (p < 0.05; Table 5).

**Table 5:** Distribution of patients' emergency department discharges The data has been represented as N, %. Also, included is the value at which the p-value is considered significant (p<0.05).

	Pre-pandemic Period n = 13187 (%)	Pandemic Period n = 3543 (%)		p
Emergency Department Discharges			x2 = 547,901	0.001
Discharge	11938 (90.5)	2695 (76.1)		
Hospitalization	937 (7.1)	687 (19.4)		
Referral	275 (2.1)	143 (4.0)		
Exitus	37 (0.3)	18 (0.5)		

## Discussion

The COVID-19 pandemic recently became humanity’s main challenge, with the WHO declaring it a global pandemic in January 2020. A decrease in hospital emergency room admissions was reported worldwide during this time [7]. A decrease was observed in the number of PES and emergency department admissions. Our retrospective study observed that the number of patients admitted to PES decreased by 73.13% with the pandemic. The reasons for this decrease include the measures taken in our country to prevent the spread of COVID-19, such as curfews, hesitation to head to the emergency department for fear of being infected, and the closure of educational institutions. It is additionally thought that the increased use of masks, gloves, and attention to hand hygiene in society during the pandemic reduced infectious disease rates. There was also a significant decrease in the rate of nosocomial (healthcare-associated) infections, which threaten public health, during the pandemic compared to the pre-pandemic period [8]. In articles similar to our study comparing the pre-pandemic and pandemic periods in pediatric emergency department admissions, it was observed that patient admissions decreased by 59% in Greece, 68.9% in Italy, and 51% in the USA during the pandemic period [7,9,10].

It was also observed that the average age of patients admitted to pediatric emergency departments increased during the pandemic: instead of in-school-age children, the most common PES admissions were in the adolescent age group (Figure 1). Still, there was a significant decrease in the number of emergency department admissions in all age groups during the pandemic period. The reason for this decrease, especially in children under the age of six and school-age children in the 7-12 age group, maybe the decrease in infectious diseases commonly seen in these age groups due to restrictions on communal areas such as schools, curfews, paying attention to hygiene, and reducing social contact. In a study conducted in the USA, it was similarly observed that admissions of patients under the age of 14 decreased by 70%, especially in the first weeks of the pandemic, with the start of processes such as school closures and curfews [11].

When a comparison was made according to nationality, type of admission, and triage classification, although the number of Turkish citizen and foreign national patient admissions decreased significantly during the pandemic period, there was a proportional increase in the PES admissions of foreign national patients during the pandemic period. It can be assumed that foreign patients are more adequate at implementing and understanding the measures and restrictions implemented during the pandemic [12]. In our study, we additionally observed a significant decrease in admissions at all times of the day. The highest number of admissions occurred in the pre-pandemic period and in the "16:00-00:00" interval during the pandemic period. A similar study observed that most of the admissions occurred outside of working hours; it was thought that the number of PES admissions increased after 16:00 because parents wanted to bring their children to the hospital after work to avoid waiting in line at the polyclinic and to receive health services quickly by admitting them to the emergency department [13].

When we compared the types of patient admission to the PES, we observed that although the number of admissions decreased during the pandemic period in both types of admission, there was an increase in the rate of admission via ambulance. Similarly, a study conducted in Taiwan observed that the rate of admission to the emergency department by ambulance increased from 12.63% to 17.91% during the pandemic [14]. Another study conducted in Ireland observed that while the number of PES admissions by ambulance was 6% in the pre-pandemic period, it increased to 7% and 8%, respectively, during the first two months of the pandemic [15]. Patients were thought to be more likely to arrive by ambulance due to restrictions during the pandemic. At the same time, the increase in morbidity and mortality due to patients not arriving at the hospital on time due to the risk of infection transmission was thought to be another reason for the increase in ambulance referrals and admissions.

Moreover, there was a significant difference in the triage evaluation of patients between the pre-pandemic period and the pandemic period. Although the number of patients in all triage classifications decreased during the pandemic period, it was observed that the proportion of patients in the triage classification defined as "Green" decreased significantly, while the admission rates in the "Red" and "Yellow" triage classifications increased. An Italian study similarly showed a decrease in patients in the "Green" and "White" triage classifications who did not require urgent medical intervention [16]. Among the reasons for the significant decrease in patients identified as "Green" during the pandemic period is the lower incidence of infectious upper respiratory tract diseases, which are common in childhood; the decrease in patients who want to receive faster health care in emergency departments only for examination and general examination; and the decrease in unnecessary admissions to the emergency department due to COVID-19 restrictions. Among the reasons for the increase in the proportion of patients in the "Yellow" and "Red" triage classifications may be the fact that patients with increased risk of morbidity and mortality were admitted after their complaints progressed, as well as referrals from external centers to health care institutions such as the third-level health institution where our study was conducted.

Furthermore, during the pandemic period, the decrease in admissions of chronic and follow-up patients (e.g., thalassemia, G6PD deficiency, sickle cell anemia, hematologic malignancy) associated with the diagnoses in the "Hematologic-Oncologic System Diseases" diagnosis group was less than in other disease groups. There has also been a relative increase in the incidence of patients with new diagnoses of "Hematologic-Oncologic System Diseases" and follow-up. Patients with a recurrent need for blood transfusion, replacement therapy, and hydration were similarly observed to have PES admissions during the pandemic. This may suggest that there were no unnecessary admissions before the pandemic in this disease group. A study conducted in Romania observed a 10% decrease in the group of oncologic patients admitted as outpatients during the pandemic period, which was again attributed to the measures taken due to the pandemic, which led to a decrease in the number of outpatients [17].

The number of patients diagnosed with metabolic disorders in the diagnosis group "Endocrine and Metabolic Diseases" decreased during the pandemic period. The incidence rate of "Endocrine and Metabolic Diseases" increased due to a significant decrease in the diagnosis group "Respiratory Diseases.” This suggests that pandemic measures have had relatively little impact on metabolic disease admissions.

There was no decrease in the number of patients evaluated in our PES with diagnoses in the "Psychiatric Diseases" diagnostic group. Still, there was an increase in the number and rate of mood disorders. A separate Turkish study identified that the dramatic impact of the COVID-19 pandemic had also affected children and young people and that children, just like adults, faced difficulties due to fear, uncertainty, and social constraints during this period. Likewise, it is emphasized that it is important for patients with chronic psychiatric disorders to use their medication regularly and ensure their medication supply [18]. The increase in the admissions of those with psychiatric diseases to our institution supports this study.

There was also an increase in the number of patients evaluated and admitted with the diagnosis of "Cerebral Palsy and Other Paralytic Diseases" in the "Nervous System Diseases" diagnostic group. It is thought that patients requiring regular follow-up, such as those with cerebral palsy, were admitted to PES during the pandemic period due to restrictions and difficulties in accessing services such as physical therapy. A study conducted in India similarly revealed that children with cerebral palsy were greatly affected by the COVID-19 pandemic, with deterioration in their functions, progression of deformities, inability to access regular physical therapy/orthotics services, and the development of behavioral problems [19].

Notably, the number and rate of admissions decreased in patients evaluated with the diagnosis of "Middle Ear Diseases" in the diagnosis group "Eye- and ENT-Related Diseases.” Admissions of those with middle ear diseases such as otitis media, which are associated with agents associated with respiratory diseases, significantly decreased during the pandemic period. A study conducted in Italy observed that the incidence of acute otitis media decreased from 5.3% to 1.6% with the restrictions implemented during the pandemic period [20]. With the COVID-19 pandemic, it is thought that global attention to masks, hygiene, and cleanliness has led to a decrease in diseases caused by respiratory infectious agents and fewer complications related to these diseases.

In the "Circulatory System Diseases" diagnostic group, an increase in the number and frequency of admissions of patients evaluated for myocarditis is noteworthy. Myocarditis is a disease in which inflammatory infiltration occurs in the heart, causing myocardial damage. The etiology of myocarditis in children includes viral and infectious causes. COVID-19-associated myocarditis is also present in the etiology [21]. In addition, children with COVID-19 can develop arrhythmias, myocarditis, and shock. This clinical condition is called multisystemic inflammatory syndrome (MIS-C) [22]. A study in China similarly observed that influenza-induced respiratory diseases decreased significantly during the pandemic [23]. Meanwhile, a study in Italy found a significant decrease of 79.34% on average in respiratory syncytial virus (RSV)-induced respiratory diseases compared to the last three years [24]. The lower incidence of agents such as influenza and RSV is thought to reduce respiratory infections and related complications.

Contrastingly, there was an increase in the incidence of diseases in the diagnosis group "Digestive System Diseases.” It is thought that there was a relative increase in patients presenting with gastrointestinal system diseases due to the significant decrease in respiratory diseases during the pandemic. In a study conducted in China, it was observed that the admission rate for "Digestive System Diseases" increased from 23.9% to 33.3% with the pandemic. In diagnoses affecting the stomach and duodenum, such as acute gastritis, both the number of patients and the rate of admission increased in line with our findings [25]. An Israeli study showed that the prevalence of obesity, especially in children between the ages of 2 and 5.9, increased compared to the average of the two years before the pandemic [26]. However, an increase in esophageal, gastric, and duodenal diseases was observed in this group in terms of both number and proportional increase, which may be attributed to the increase in time spent at home due to the pandemic and consequential changes in eating habits.

In the diagnosis groups of "Obstetric and Gynecological Diseases" and "Psychiatric Diseases,” the number and rate of patients admitted during the pandemic increased. Most patients in the "Obstetric and Gynecologic Diseases" diagnostic group admitted during the pandemic were child-aged pregnant women, a reality for the world and our country. It was the most common diagnosis in all disease groups. Studies in different countries have shown that child marriage rates have increased with the pandemic [27]. It is thought that pediatric and pregnant patient admissions increased because risky patients were sent to third-level health institutions during the pandemic. However, it is worth noting that the increased number of pregnancies at this age may have been due to difficulty accessing health institutions to terminate pregnancy due to the pandemic control measures taken.

Notably, while the emergency department discharge rate decreased during the pandemic, the hospitalization rate increased. All emergency department discharges decreased during the pandemic period. A study conducted in Israel similarly identified a decrease in the number of patient admissions and an increase in hospitalization rates [28]. Among the reasons for this include the fact that patients who could be diagnosed and treated in primary health care services were admitted to the emergency departments of tertiary health institutions such as our institution; in addition, respiratory tract infections and complications, which are common in childhood, became less common in response to measures such as hygiene, masks, social distancing, and curfews were taken with the pandemic [29]. In pediatric emergency department admissions, there was a decrease in the proportion of green-area patients. In contrast, the proportion of yellow- and red-area patients with a great need for inpatient treatment increased. Identifying patients with urgent medical needs and epidemiological data obtained through similar studies will guide plans to reduce congestion and provide effective health services. This study has potential limitations. The pandemic period, especially the hesitation of patients with chronic diseases to apply to the hospital, may have prevented the emergence of the real pediatric emergency department patient profile. The late presentation of patients with poor general conditions to the hospital may have caused differences in the number of hospitalizations and referrals.

## Conclusions

Since the beginning of the COVID-19 pandemic, healthcare services worldwide have been affected. The data obtained in our study indicate a significant decrease in the number of PES admissions during the pandemic period. This is in line with the literature, which demonstrates a decrease in the number of PES admissions worldwide during the pandemic. The COVID-19 pandemic, which led to varying effects on emergency health services worldwide, reduced the incidence of diseases that manifest themselves with infectious agents, such as respiratory infections, due to the safety measures taken in our country. A striking change was observed in pediatric emergency department presentation habits. As seen in the literature, mask, hygiene, and social distancing restrictions led to a decrease in infectious diseases. One of the reasons for the significant decrease in hospital admissions was the fear of being infected with COVID-19, especially in collaborative environments such as hospitals.

The change in PES habits with the pandemic highlights the importance of conducting more comprehensive epidemiological studies in terms of more efficient and effective use of pediatric emergency health services in Turkey. Comprehensive epidemiological studies on this subject would shed light on future health policies in our country, mainly due to the high number of admissions, unnecessary admissions to emergency departments for common problems, and the burden of health services in emergency departments.

## References

[1] (2022). Current Turkish Dictionary. https://sozluk.gov.tr/.

[2] T.C. Acil Sağlık Hizmetleri Yönetmeliği (2022). Emergency health services regulation [Website in Turkish]. https://dosyamerkez.saglik.gov.tr/Eklenti/19158/0/acil-saglik-hizmetleri-yonetmeligi-11052000-tarihli-24046-sayili-resmi-gazetepdf.pdf.

[3] Özel G (2019). Determination of factors affecting pediatric emergency department density [Website in Turkish]. Adıyaman; Adıyaman Üniversitesi Tıp Fakültesi.

[4] (2022). Current status and recommendations of pediatric emergency medical services in Turkey and the world [Article in Turkish]. http://cayd.org.tr/files/turkiye-ve-dunyada-cocuk-acil-tip-hizmetleri-raporu-mV.pdf.

[5] Temizkan RC, Büyük N, Kılıçarslan Ö, Ankaralı H, Kocabay K (2019). Characteristics of patients admitted to the pediatric emergency department of a Medical Faculty Hospital [Article in Turkish]. Anatol Clin J Med Sci.

[6] Cucinotta D, Vanelli M (2020). WHO declares COVID-19 a pandemic. Acta Biomed.

[7] Kostopoulou E, Gkentzi D, Papasotiriou M, Fouzas S, Tagalaki A, Varvarigou A, Dimitriou G (2022). The impact of COVID-19 on paediatric emergency department visits. A one-year retrospective study. Pediatr Res.

[8] İpek S, Şahin A, Gungor S, Yurttutan S, Güllü UU, Inal S, Demiray Ş (2022). Nosocomial infections in non-COVID-19 pediatric patients prior to and during the pandemic in a pediatric intensive care unit. Cureus.

[9] Valitutti F, Zenzeri L, Mauro A (2020). Effect of population lockdown on pediatric emergency room demands in the era of COVID-19. Front Pediatr.

[10] Radhakrishnan L, Leeb RT, Bitsko RH (2022). Pediatric emergency department visits associated with mental health conditions before and during the COVID-19 pandemic - United States, January 2019-January 2022. MMWR Morb Mortal Wkly Rep.

[11] DeLaroche AM, Rodean J, Aronson PL (2021). Pediatric emergency department visits at US children’s hospitals during the COVID-19 pandemic. Pediatrics.

[12] Şahin D, Alramazanoğlu BO (2021). Investigation of the socio-demographic characteristics, admission and death rates of patients admitted to a pandemic hospital due to COVID-19 [Article in Turkish]. Int J Bus Sci Appl.

[13] Anıl M, Anıl AB, Köse E, Akbay S, Helvacı M, Aksu N (2014). The evaluation of the patients admitted to the pediatric emergency department in a training and research hospital [Article in Turkish]. J Pediatr Emerg Intensive Care Med.

[14] Tsai LH, Chien CY, Chen CB (2021). Impact of the coronavirus disease 2019 pandemic on an emergency department service: experience at the largest tertiary center in Taiwan. Risk Manag Healthc Policy.

[15] McDonnell T, Nicholson E, Conlon C, Barrett M, Cummins F, Hensey C, McAuliffe E (2020). Assessing the impact of COVID-19 public health stages on paediatric emergency attendance. Int J Environ Res Public Health.

[16] Barbiellini Amidei C, Buja A, Bardin A (2021). Pediatric emergency department visits during the COVID-19 pandemic: a large retrospective population-based study. Ital J Pediatr.

[17] Crisan C, Cainap C, Deac A (2021). Decrease of oncological patients' hospital visits during Covid-19 pandemic; the experience of a tertiary Romanian centre. J Clin Oncol.

[18] Pilan BŞ, Erermiş S, Çalışan R (2021). Adaptation process and disease symptoms of children and young people under psychiatric follow-up with chronic medical diseases during pandemic days [Article in Turkish]. Aegean Med J.

[19] Bhaskar AR, Gad MV, Rathod CM (2022). Impact of COVID pandemic on the children with cerebral palsy. Indian J Orthop.

[20] Torretta S, Cantoni B, Bertolozzi G (2021). Has otitis media disappeared during COVID-19 pandemic? A fortuitus effect of domestic confinement. J Clin Med.

[21] Williams JL, Jacobs HM, Lee S (2023). Pediatric myocarditis. Cardiol Ther.

[22] Güllü UU, Güngör Ş, İpek S, Yurttutan S, Dilber C (2021). Predictive value of cardiac markers in the prognosis of COVID-19 in children. Am J Emerg Med.

[23] Zheng L, Qi J, Wu J, Zheng M (2022). Changes in influenza activity and circulating subtypes during the COVID-19 outbreak in China. Front Med (Lausanne).

[24] Vittucci AC, Piccioni L, Coltella L (2021). The disappearance of respiratory viruses in children during the COVID-19 pandemic. Int J Environ Res Public Health.

[25] Chen YJ, Chen CY, Lee EP, Huang WY, Wu HP (2022). Influence of the domestic COVID-19 pandemic on the pediatric emergency department. Front Med (Lausanne).

[26] Dubnov-Raz G, Maor S, Ziv-Baran T (2022). Pediatric obesity and body weight following the COVID-19 pandemic. Child Care Health Dev.

[27] Okeke SR, Idriss-Wheeler D, Yaya S (2022). Adolescent pregnancy in the time of COVID-19: what are the implications for sexual and reproductive health and rights globally?. Reprod Health.

[28] Erlichman M, Zalut T, Schwartz S, Weiser G (2021). The ongoing indirect effect of the COVID-19 pandemic on a pediatric emergency department. PLoS One.

[29] Diesner-Treiber SC, Voitl P, Voitl JJ (2021). Respiratory infections in children during a COVID-19 pandemic winter. Front Pediatr.

